# Major incident triage and the implementation of a new triage tool, the MPTT-24

**DOI:** 10.1136/jramc-2017-000819

**Published:** 2017-10-21

**Authors:** James Vassallo, J E Smith, L A Wallis

**Affiliations:** 1 Division of Emergency Medicine, University of Cape Town, Cape Town, South Africa; 2 Institute of Naval Medicine, Gosport, UK; 3 Emergency Department, Derriford Hospital, Plymouth, UK; 4 Academic Department of Military Emergency Medicine, Royal Centre for Defence Medicine (Research & Academia), Medical Directorate, Birmingham, UK

**Keywords:** triage, mass casualty incidents, life-saving interventions, major incidents

## Abstract

**Introduction:**

The Modified Physiological Triage Tool (MPTT) is a recently developed primary triage tool and in comparison with existing tools demonstrates the greatest sensitivity at predicting need for life-saving intervention (LSI) within both military and civilian populations. To improve its applicability, we proposed to increase the upper respiratory rate (RR) threshold to 24 breaths per minute (bpm) to produce the MPTT-24. Our aim was to conduct a feasibility analysis of the proposed MPTT-24, comparing its performance with the existing UK Military Sieve.

**Method:**

A retrospective review of the Joint Theatre Trauma Registry (JTTR) and Trauma Audit Research Network (TARN) databases was performed for all adult (>18 years) patients presenting between 2006–2013 (JTTR) and 2014 (TARN). Patients were defined as priority one (P1) if they received one or more LSIs. Using first recorded hospital RR in isolation, sensitivity and specificity of the ≥24 bpm threshold was compared with the existing threshold (≥22 bpm) at predicting P1 status. Patients were then categorised as P1 or not-P1 by the MPTT, MPTT-24 and the UK Military Sieve.

**Results:**

The MPTT and MPTT-24 outperformed existing UK methods of triage with a statistically significant (p<0.001) increase in sensitivity of between 25.5% and 29.5%. In both populations, the MPTT-24 demonstrated an absolute reduction in sensitivity with an increase in specificity when compared with the MPTT. A statistically significant difference was observed between the MPTT and MPTT-24 in the way they categorised TARN and JTTR cases as P1 (p<0.001).

**Conclusions:**

When compared with the existing MPTT, the MPTT-24 allows for a more rapid triage assessment. Both continue to outperform existing methods of primary major incident triage and within the military setting, the slight increase in undertriage is offset by a reduction in overtriage. We recommend that the MPTT-24 be considered as a replacement to the existing UK Military Sieve.

**Key messages**

- The Modified Physiological Triage Tool (MPTT) was derived on a military cohort using logistic regression and outperforms all existing triage tools at predicting the need for life-saving intervention in both military and civilian populations.
- Increasing the upper respiratory rate threshold to 24 (MPTT-24) allows for a reduction in the time required to use the triage tool.
- Using the Alert; responds to Verbal stimulus; responds to Painful stimulus; Unresponsive (AVPU) scale as supposed to the GCS to measure conscious level will enable the MPTT-24 to be used by a greater number of personnel, increasing its applicability.
- Performance of the MPTT-24 is largely unchanged from the MPTT, and it clinically and statistically outperforms the existing UK Military Sieve at predicting the need for life-saving intervention.
- We recommend that the MPTT-24 be considered as an alternative to the existing UK Military Sieve for the purposes of primary major incident triage in the military setting.

## Introduction

Triage is the process of prioritising patients on the basis of their clinical acuity and is a key principle of effective major incident management.^1^ Within the UK, existing military and civilian doctrine utilises a two-stage approach to triage with primary and secondary triage being performed. Primary triage is a quick assessment of the patient, conducted at the scene and is frequently performed in difficult settings. For it to be effective, it must be rapid, reliable and reproducible, irrespective of the provider using it.^1^

The UK Military Sieve and National Ambulance Resilience Unit Sieve are the algorithms used by the Defence Medical Services and Ambulance Services, respectively, for primary major incident triage.^2 3^ Utilising simple physiological assessments, patients are categorised into one of three categories with priority one the most urgent. Secondary triage takes place in a more permissive environment, such as at a casualty clearing station or at the hospital entrance. Unlike primary triage, it is a more thorough assessment of the patient, frequently performed by more experienced and senior clinicians. If needed, it allows for the refinement of the triage category allocated during the primary triage process prior to treatment or admission to hospital.^1^

A number of studies have shown that existing methods of triage have limited accuracy at predicting the need for life-saving intervention in both the military and civilian environments.^4^ Derived specifically for this purpose, the Modified Physiological Triage Tool (MPTT) has shown the greatest sensitivity for predicting the need for life-saving intervention, with the lowest rates of undertriage and acceptable levels of overtriage in both military and civilian populations.^5–7^

Respiratory Rate (RR) and Glasgow Coma Scale (GCS) form key components of the MPTT and can both be time consuming to accurately measure, with significant inter-rater reliability being described previously.^8^ We propose to modify the MPTT by increasing the upper respiratory rate threshold to 24 breaths per minute (MPTT-24), allowing providers to more easily do a 15 second RR count and multiply by four (or 10 seconds and multiply by six), thereby potentially halving the time currently required to use the MPTT. In addition, we have adopted the Alert; responds to Verbal stimulus; responds to Painful stimulus; Unresponsive (AVPU) scale for the purposes of the conscious level assessment, replacing the existing GCS<14 assessment.^9 10^

Accepting a more pragmatic approach—with a threshold RR which is easily calculated within a shorter time frame—may change the test characteristics of the MPTT. The aim of this study was to conduct a feasibility analysis of the proposed MPTT-24 and compare its test characteristics with both the original MPTT and the existing UK Military Sieve.

## Materials and methods

A retrospective review of the Joint Theatre Trauma Registry (JTTR) and Trauma Audit Research Network (TARN) databases was performed for all adult (>18 years) patients presenting between 2006–2013 (JTTR) and 2014 (TARN).

The JTTR holds data on all seriously injured patients treated by UK Defence Medical Services in the deployed setting with continuous data available from 2003. The default entry criterion was a patient who triggered trauma team activation, but this was expanded in 2007 to include all patients with trauma who were returned to the Royal Centre for Defence Medicine for definitive treatment.^5 11^ Established in 1988, TARN is the largest trauma database in Europe, collecting data from all trauma receiving hospitals in England and Wales on patients with moderate to severe injuries and contains data from point of injury through to discharge. TARN inclusion criteria include hospital admission >3 days, admission to critical care or death in hospital.^12 13^ Patients declared dead at scene and not conveyed to hospital are not included in the database. Patients were assumed to be non-ambulant due to the nature of the TARN database and its inclusion criteria.^6^

**Figure 1:**
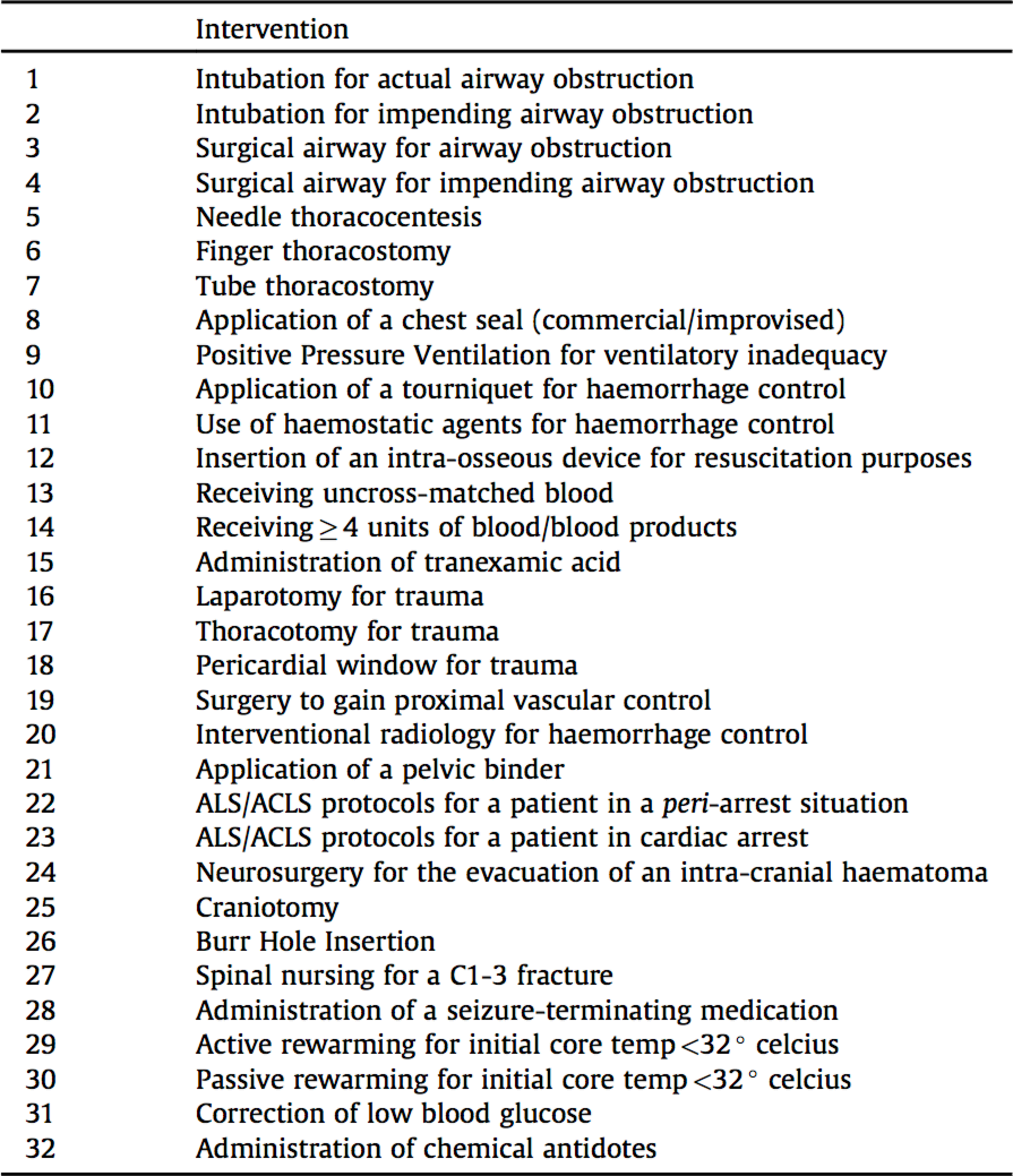
Life-saving interventions defining the priority one patient. ACLS, advanced cardiovascular life support.

**Figure 2:**
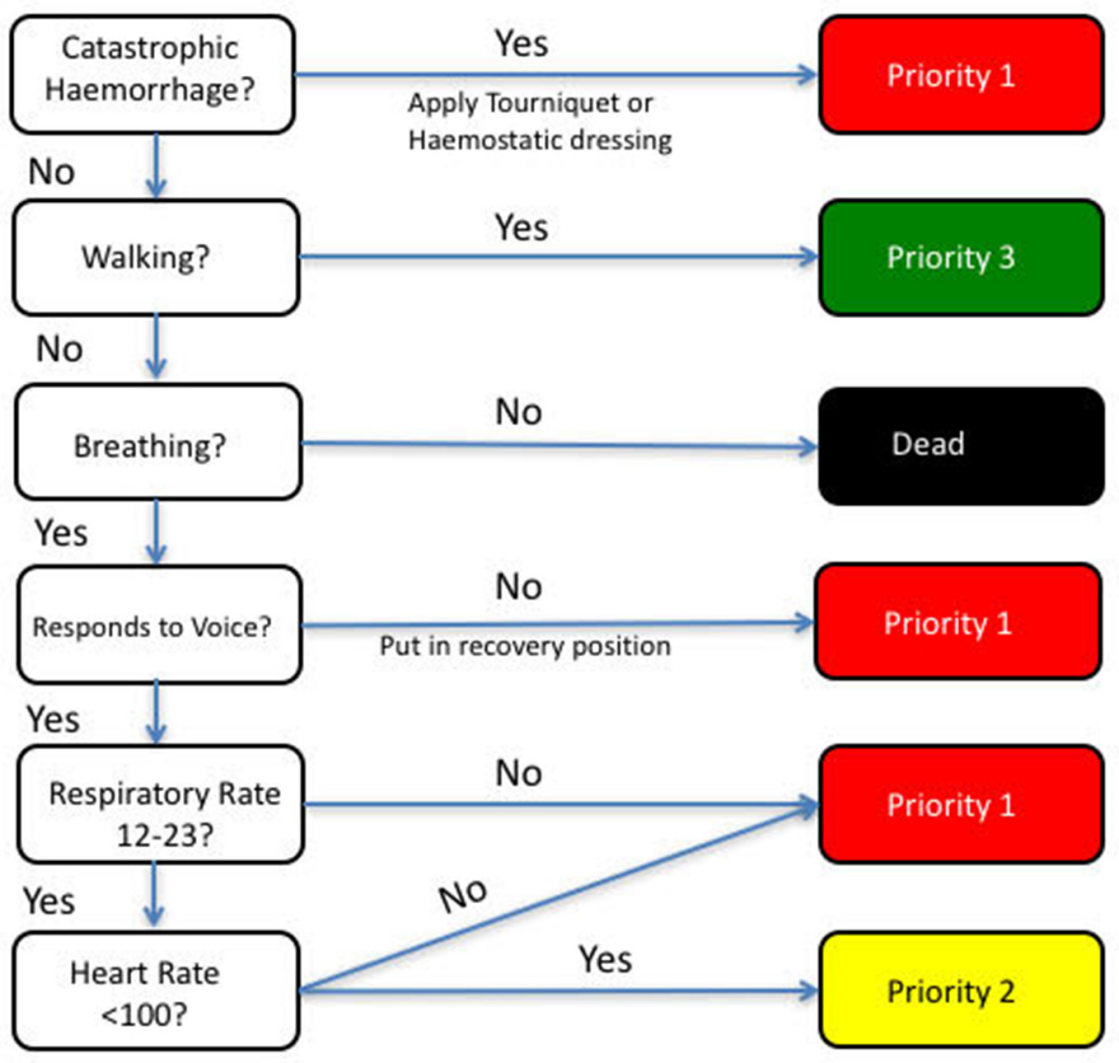
Modified Physiological Triage Tool (MPTT)-24 algorithm with increased respiratory rate upper threshold (≥24), conscious level assessment using Alert; responds to Verbal stimulus; responds to Painful stimulus; Unresponsive (AVPU scale) and the additional assessment for external catastrophic haemorrhage. Vassallo 2017. CC BY 4.0.

Patients were defined as priority one (P1) if they had received one or more life-saving interventions from a previously defined list, derived through international consensus of experts involved in major incident management (Figure 1).^14^ Using first recorded hospital RR in isolation, the sensitivity and specificity of the ≥24 breaths per minute threshold were compared with the existing threshold (≥22 breaths per minute) at predicting P1 status. Patients were then categorised as P1 or not-P1 by the MPTT, the MPTT-24 (Figure 2) and the UK Military Sieve. A McNemar test was used to determine statistical significance between the triage tools.

## Ethics statement

The use of the JTTR was approved by the Medical Directorate, Royal Centre for Defence Medicine. Additionally, as part of a larger programme of work, this study received ethical approval from the Human Research Ethics Committee of the University of Cape Town, the primary academic institution of the lead author (reference 285/2013).

## Results

Basic study characteristics are shown in table 1. In both populations, the increased threshold in RR in isolation was associated with an absolute reduction in sensitivity (TARN 11.4%, JTTR 13.5%) and an increase in specificity (TARN 8.9%, JTTR 13.8%). An increase in OR and positive predictive value was observed when using a higher RR for both TARN and JTTR (table 2).

**Table 1:** Study characteristics

	JTTR (2006–2013)	TARN (2006–2014)
Number of cases	3654	127 233
Male N, %	3593 (98.3%)	70 747 (55.6%)
Age, median (IQR)	24 (21–29)	61.4 (43.1–80.0)
ISS, median (IQR)	5 (2–16)	9 (9–16)
Mortality	2.1%	5.7%
Mechanism of injury N,%	Explosive 2012, 55.1% GSW 1252, 34.3%	Fall <2 m 18 141, 14.3% RTC 27 915, 21.9%
Injured body region N,%	Lower limb 1317, 36.0% Upper limb 593, 16.2%	Limb 43 989, 34.6% Head 24 732, 19.4%
Priority One N,%	1738, 47.6%	24 791, 19.5%

GSW, Gun shot wound; JTTR, Joint Theatre Trauma Registry; RTC, Road traffic collision; TARN, Trauma Audit Research Network.

**Table 2:** Performance analysis of test characteristics for RR (≥22 and 24 thresholds), MPTT, MPTT-24 and the existing UK Military Sieve

	Sensitivity	Specificity	OR	PPV
**JTTR**				
RR≥22	48.7% (45.5–51.9)	68.2% (65.1%–71.2%)	2.04 (1.69–2.45)	61.1% (65.2–71.2)
RR≥24	35.2% (32.2–38.4)	82.0% (79.4%–84.4%)	2.48 (2.01–3.07)	66.7% (62.5–70.7)
MPTT	69.9% (67.7–72.0)	65.3% (63.2–67.4)	4.37 (1.90–5.02)	64.8% (62.7–67.0)
MPTT-24	66.7% (64.5–68.9)	69.9% (67.8–71.9)	4.65 (2.02–5.34)	67.0% (64.7–69.1)
UK Military Sieve	43.2% (40.9–45.6)	93.7% (92.5–94.7)	11.29 (9.18–13.88)	86.1% (83.6–88.4)
**TARN**				
RR≥22	47.5% (46.7–48.3)	73.9% (73.6–74.3)	2.56 (2.47–4.16)	35.6% (35.0–36.3)
RR≥24	36.1% (35.4–36.9)	82.8% (82.5–83.2)	2.73 (1.31–2.84)	39.0% (38.3–39.8)
MPTT	57.8% (56.9–58.2)	71.5% (71.3–71.8)	3.41 (3.31–3.51)	32.9% (32.4–33.2)
MPTT-24	53.5% (52.9–54.1)	74.8% (74.6–75.1)	3.43 (3.33–3.53)	34.0% (33.4–34.5)
UK Military Sieve	28.0% (27.5–28.6)	94.1% (93.9–94.2)	6.17 (5.94–6.41)	53.3% (52.5–54.2)

JTTR, Joint Theatre Trauma Registry; RR, Respiratory Rate; MPTT, Modified Physiological Triage Tool; PPV, positive predictive value; TARN, Trauma Audit Research Network.

When incorporated into the MPTT-24, an absolute reduction in sensitivity was observed (TARN 8.9%, JTTR 3.2%) with an increase in specificity. There was a statistically significant difference between the MPTT and MPTT-24 in the way they categorised TARN and JTTR cases as P1 (p<0.001).

The MPTT-24 demonstrated a statistically significant (p<0.001) increase in sensitivity (TARN 25.5%, JTTR 23.5%) over the existing UK Military Sieve in its ability to identify those in need of a life-saving intervention.

## Discussion

In this study, we have demonstrated that pragmatic modifications to the MPTT, in the form of the MPTT-24, can be implemented while maintaining comparable performance at predicting the need for life-saving intervention in both civilian and military trauma registry populations. With these modifications, the MPTT-24 continues to outperform the existing UK Military Sieve.

In keeping with the existing UK Military Sieve,^2^ we have included an assessment of catastrophic external haemorrhage in the MPTT-24. While experience of such injuries is likely to be limited in day-to-day civilian trauma care, we note previous European terrorist major incidents (Paris 2015 and London 2007) where the demand for tourniquets was high.^15^ In the context of an ongoing Marauding Terrorist Firearms Attack, the ability to provide treatment will be limited; controlling catastrophic external haemorrhage through tourniquets or haemostatic dressings may help to preserve life until the incident becomes more permissive.

The MPTT assesses the patient’s conscious level using the GCS, with patients scoring 13 or lower being considered P1. While this represents the optimum threshold of conscious level at predicting need for life-saving intervention, it is not without limitations.^5^ Previous studies have demonstrated wide inter-rater reliability when using the GCS.^8^ Calculating the GCS requires familiarity and prior experience with the scale, but even then it can be time consuming. The AVPU score was designed as a rapid assessment of conscious level and is a simpler alternative to the GCS.^16 17^ A number of studies have looked at the correlation between GCS and AVPU with agreement that the division between being ‘alert’ and ‘responds to voice’ occurs at a median GCS of 13.^9 16 18^ For the purposes of simplifying the conscious level assessment in the primary triage process, we have replaced ‘GCS<14’ with ‘responds to voice’ in the MPTT-24. This pragmatic step should allow users with limited medical training to be able to use the MPTT-24 with similar results, thus increasing both its usability and applicability in the major incident setting.

A key principle of primary major incident triage is that it can be conducted rapidly and measuring the respiratory rate can be time consuming. By increasing the upper respiratory rate threshold of the MPTT, users are able to measure the respiratory rate over a 15 second period, allowing for a potential reduction in the time required to prioritise patients with the MPTT-24 by up to 15 seconds. If this reduction is applied to a theoretical scenario with 20 patients requiring triage, then up to 5 min could be saved by using the MPTT-24 rather than the MPTT. However, we acknowledge that this increased threshold is unlikely to convey any additional time benefit if users choose to measure the respiratory rate over a 30 second period.

Adopting the MPTT-24 comes at the expense of a reduction in sensitivity and therefore a higher rate of undertriage (1 - sensitivity). Clinically, this increased rate in undertriage is negligible; within the military setting, 30 genuine P1 patients would need to be assessed before an additional patient is undertriaged by the MPTT-24. Likewise, the reduction in overtriage (1 - positive predictive value)is negligible between the MPTT and MPTT-24 and needs even greater number of patients before a difference is observed.

In the civilian setting, the rate of overtriage for both the MPTT-24 and MPTT is high (66.0% and 67.1%, respectively). Although this is comparable to the overall overtriage rate following the London 7/7 attacks (64%),^19^ we acknowledge that if sustained, this level may not be tolerable in the setting of a non-developed system or rural environment.^20^ The MPTT and MPTT-24 were designed for the purposes of primary major incident triage alone and not as a replacement to secondary triage. Within the UK, patients will undergo a secondary triage process, with a review of the original triage categories and where appropriate, those initially overtriaged can be reallocated to a lower triage category at the discretion of experienced clinicians, thus reducing the overall overtriage rate.^1^

A key limitation of our study is the use of first recorded hospital physiology to calculate triage priorities. While prehospital data is recorded on both the JTTR and TARN databases, complete data are available for only 16.7% and 37.2% of military and civilian cases, respectively, making prioritisation with prehospital data unreliable. However, when complete hospital and prehospital physiology were compared in both datasets, we observed that the median and IQR were almost identical.

## Conclusion

When compared with the existing MPTT, the MPTT-24 allows for the potential for a more rapid triage assessment, while maintaining comparable performance and continuing to outperform existing methods used in the UK. Within a military setting, the slight increase in undertriage is offset by a reduction in overtriage. We recommend that the MPTT-24 be considered as a replacement to the existing UK Military Sieve for the purposes of primary major incident triage.
